# Supplementary material for: Early skin seeding regulatory T cells modulate PPARγ-dependent skin pigmentation
Source: Nat Commun. 2025 Dec 9;16:11411. doi: 10.1038/s41467-025-66229-2 (PMC12738793; doi:10.1038/s41467-025-66229-2)
Supplement: Supplementary file 1 — Supplementary Information [file 41467_2025_66229_MOESM1_ESM.pdf]

Supplementary material for

Early skin seeding regulatory T cells modulate PPARγ-dependent skin pigmentation

Authors: Inchul Cho[1,2], Hafsah Aziz[1,2,#], Jessie Z. Xu[1,2,#], Prudence PokWai Lui[1,2], Monica Sen[1,2], Boyu Xie[3], Pei-Hsun Tsai[1,2], Jie Ting Wang[1,2], Hee-Yeon Jeon[4], Jinwook Choi[5], Shahnawaz Ali[2], Niwa Ali[1,2] *

Affiliations:
[1]Peter Gorer Department of Immunobiology, King's College London, London, United Kingdom.
[2]Centre for Gene Therapy and Regenerative Medicine, King's College London, London, United Kingdom.
[3]Imperial College London, London, United Kingdom.
[4]King's College London, London, United Kingdom.
[5]Gwangju Institute of Science and Technology, Gwangju, South Korea.
#Equal contribution
*Corresponding author. Email: niwa.ali@kcl.ac.uk

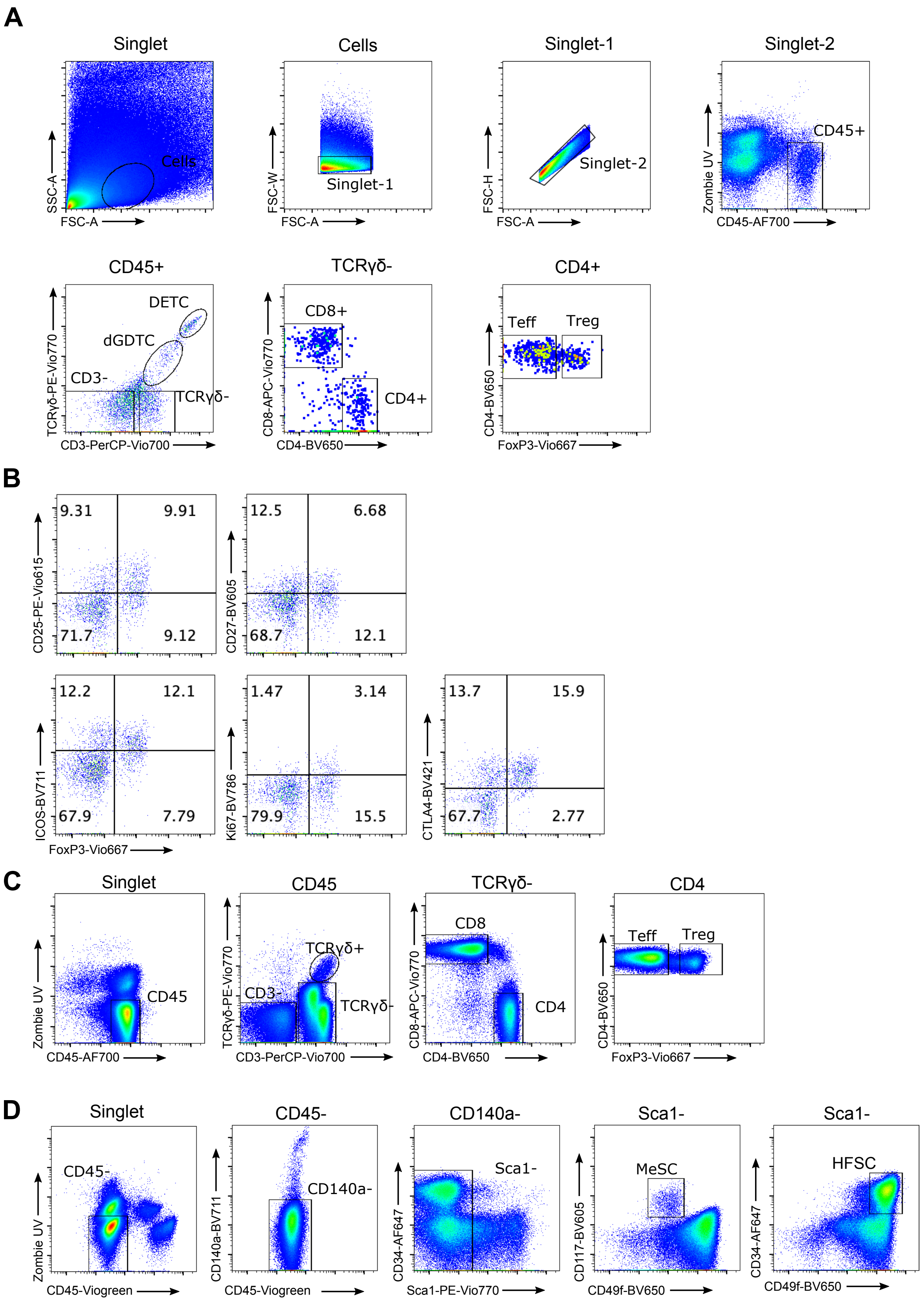

Supplementary figure 1. Flow cytometric gating strategy for analysis. A) Skin T cell gating strategy. Strict live dead and singlet inclusion criteria were applied. Immune cells were gated as $CD45^{+}$ cells. Further subsets were gated as follows: $CD3^{-}$ immune cells, $TCR\gamma\delta^{hi}$ $CD3^{+}$ DETCs (dendritic epidermal T cells), $TCR\gamma\delta^{int}$ $CD3^{+}$ dGDTCs

(dermal gamma delta T cells), TCRγδ^{-} CD3^{+} CD8^{+} T cells, TCRγδ^{-} CD3^{+} CD4^{+} FoxP3^{-} Teffs (effector T cells), and TCRγδ^{-} CD3^{+} CD4^{+} FoxP3^{+} Tregs (regulatory T cells). **B)** Representative gating of Treg phenotypic marker expression in the skin. Cells were pre-gated as CD4^{+} T cells. **C)** Gating strategy to analyse T cells in the SDLN (skin-draining lymph node). Immune cells were gated as follows: CD3^{-} immune cells, TCRγδ^{+} cells, TCRγδ^{-} CD3^{+} CD8^{+} T cells, TCRγδ^{-} CD3^{+} CD4^{+} FoxP3^{-} Teffs (effector T cells), and TCRγδ^{-} CD3^{+} CD4^{+} FoxP3^{+} Tregs (regulatory T cells). **D)** Gating strategy to identify CD45^{-} CD140a^{-} Sca1^{-} CD117^{+} MeSCs (melanocyte stem cells) and CD45^{-} CD140a^{-} Sca1^{-} CD34^{+} CD49f^{+} hair follicle stem cells (HFSCs).

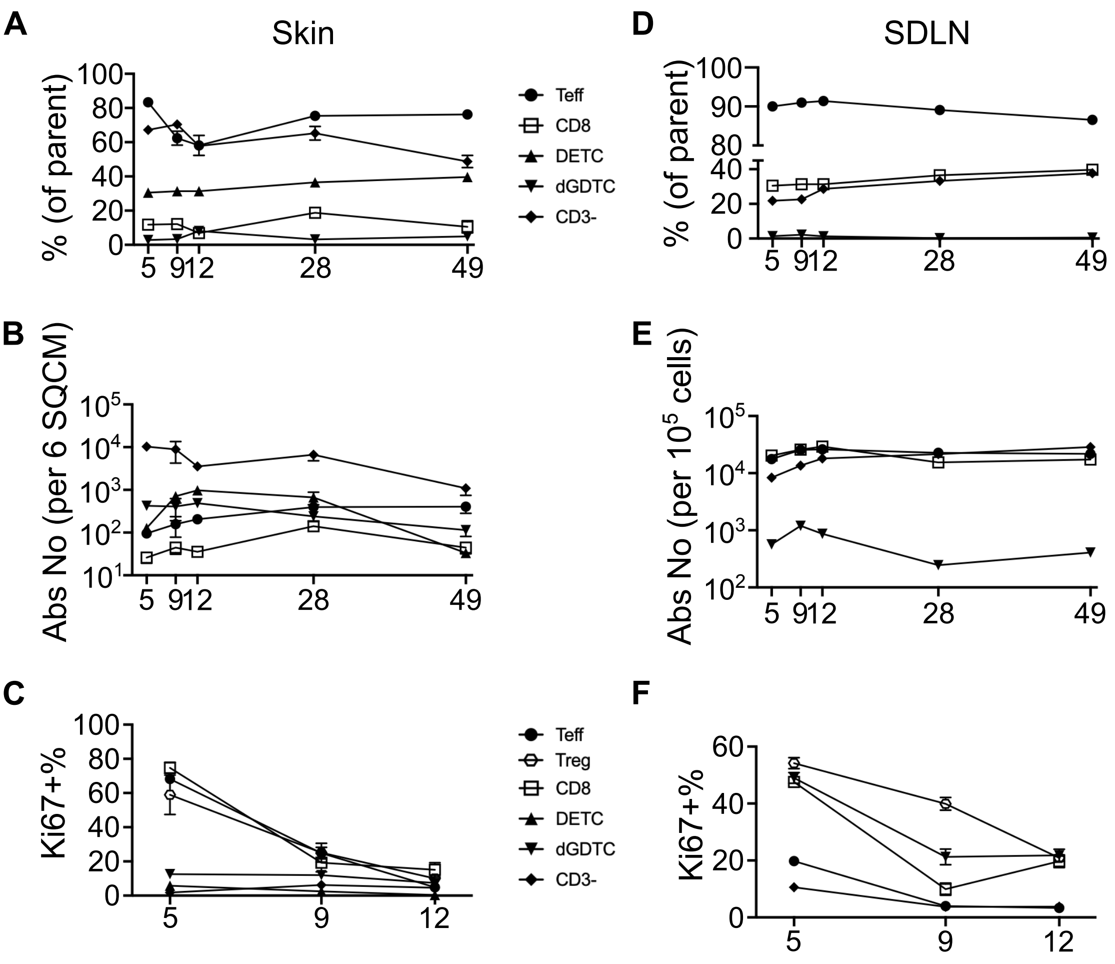

Supplementary figure 2. Flow cytometric characterisation of T cell numbers and proliferation. **A-C)** Skin-resident immune cells on postnatal day 5 (P5), P9, P12, P28 and P49. **A)** Percentage. **B)** Absolute number (per 6 cm^2 of skin)**. C)** Percentage of proliferating Ki67**⁺** subsets on P5, P9 and P12. **D-F)** SDLN-resident immune cells. **D)** Percentage. **E)** Absolute number (per 10^5 total cells). **F)** Percentage of proliferating Ki67⁺ subsets on P5, P9 and P12. Graphs show mean ± S.E.M. Data are pooled from 2 independent experiments (n=4-6 biological replicates).

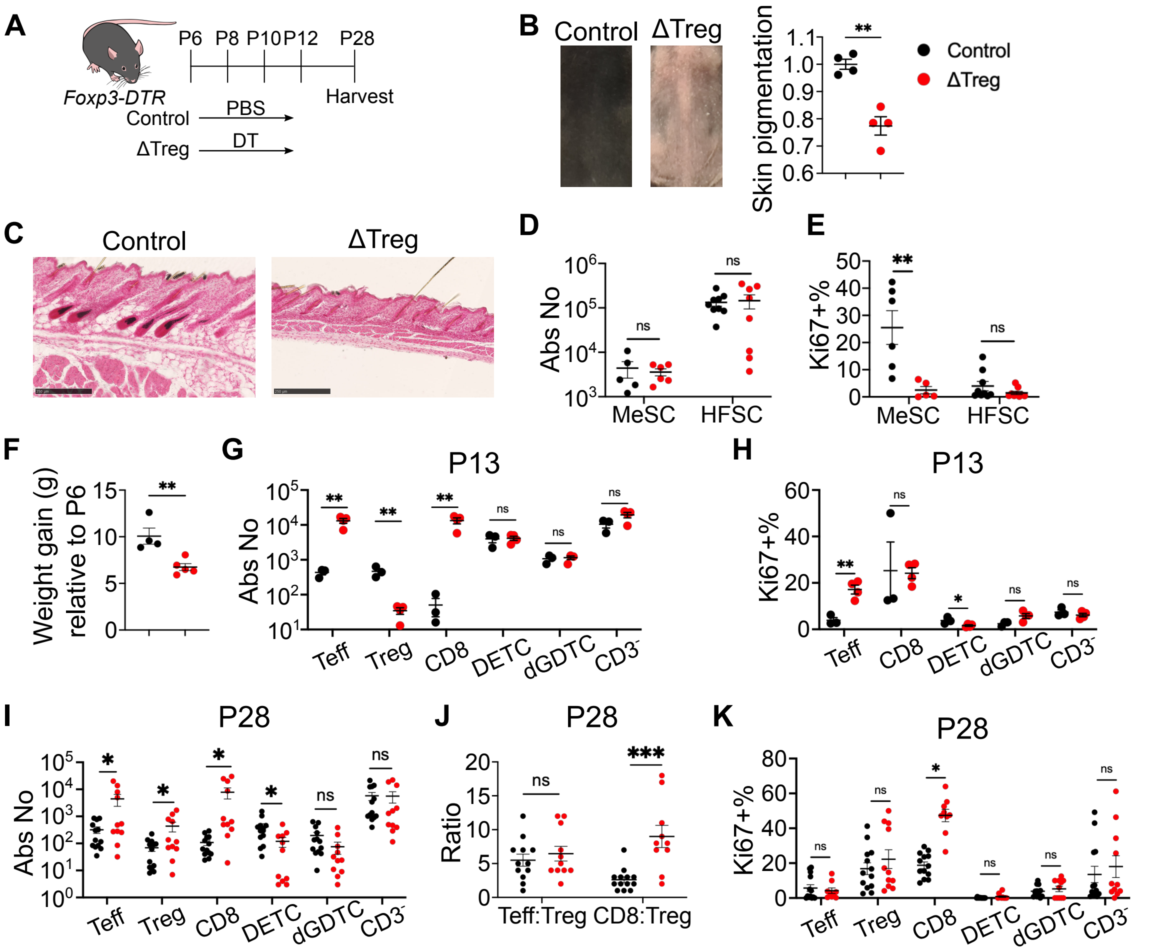

Supplementary figure 3. Four-dose DT regimen causes weight loss, inflammation, and pigment defect. A) Schematic outline of experimental timeline. (NIAID Visual & Medical Arts. (10/7/2024). Lab Mouse. NIAID NIH BIOART Source. bioart.niaid.nih.gov/bioart/281). Tregs were depleted by intraperitoneal administration of diphtheria toxin (DT) on postnatal day 6 (P6), P8, P10 and P12 (30 ng/g per injection). Skin tissues were harvested on P28. **B)** Representative images of dorsal skin on P28, and associated quantification of skin pigmentation. **C)** Fontana & Masson (F&M) staining of P28 dorsal skin. Scale bar represents 250 µm. **D-E**) Flow cytometric quantification of melanocyte stem cells (MeSCs) and hair follicle stem cells (HFSCs). **D)** Absolute number. **E)** Percentage of proliferating Ki67$^+$ cells. **F**) Weight gain from P28 to P6. **G-H)** Flow cytometric quantification of skin T cells on P13. **G)** Absolute number. **H)** Percentage of proliferating Ki67$^+$ cells. **I-K)** Flow cytometric quantification of skin T cells on P28. **I)** Absolute number. **J)** Ratio of Teff:Treg and CD8:Treg. **K)** Percentage of proliferating Ki67$^+$ subsets. Data are pooled from 4 independent experiments (n=2-4 biological replicates). Graphs show mean ± S.E.M. Unpaired t-test. ***p<0.001, **p<0.01, *p<0.05, ns p>0.05.

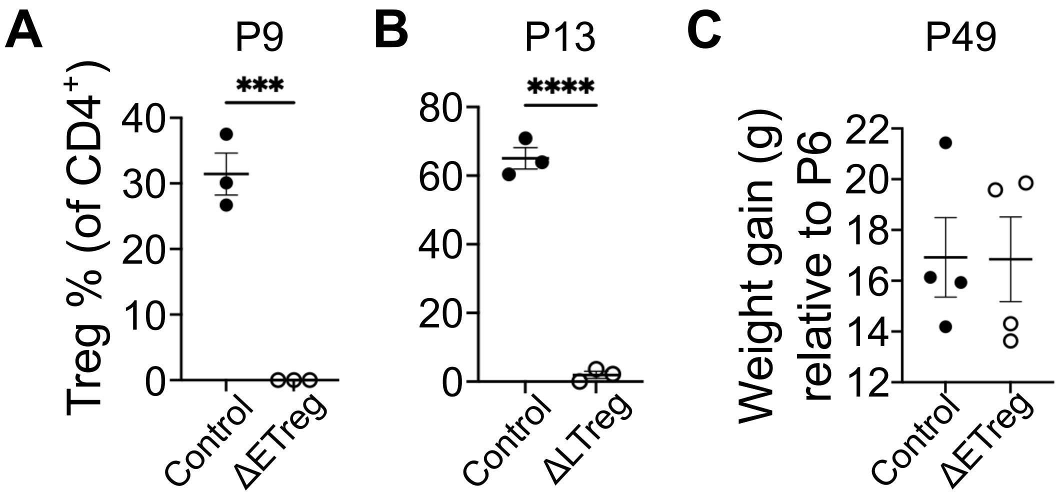

Supplementary figure 4. Transient DT injection is sufficient for skin Treg depletion. Flow cytometric profiling of Treg percentage on **A)** P9 or **B)** P13 following Treg depletion in *Foxp3-DTR* mice. **C)** Weight gain from P6 to P49 in grammes. Graphs show mean ± S.E.M. Unpaired t-test. ****p<0.0001, ***p<0.001.

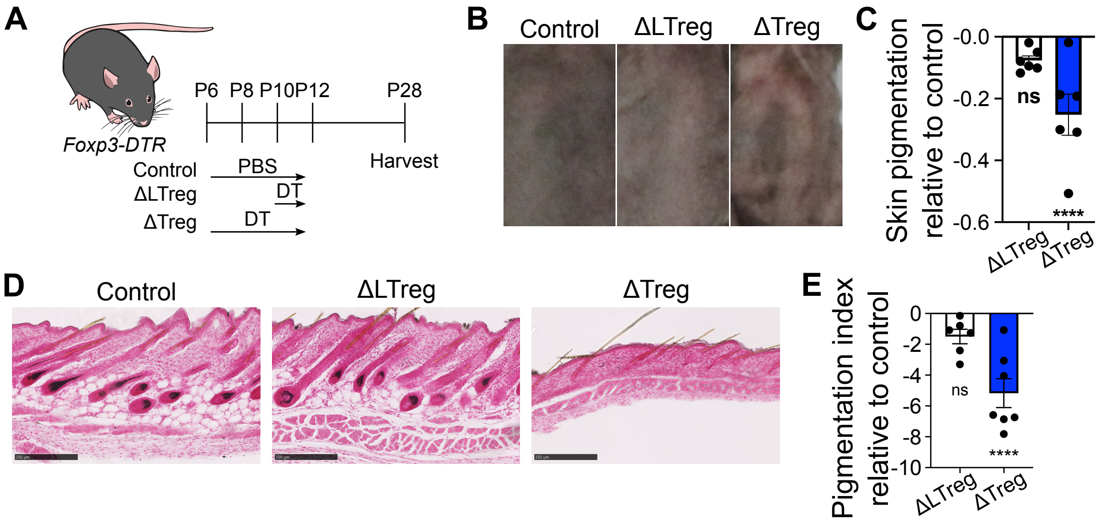

Supplementary figure 5. Late Treg depletion does not affect skin pigmentation. A) Timeline of experiment. (NIAID Visual & Medical Arts. (10/7/2024). Lab Mouse. NIAID NIH BIOART Source. bioart.niaid.nih.gov/bioart/281). Intraperitoneal injections of PBS were performed on P6, P8, P10, and P12 (Control), DT on P10 and P12 (ΔLate Tregs, ΔLTreg) and on P6, P8, P10 and P12 (ΔTreg). Mice were sacrificed on P28. **B)** Representative shaved dorsal skin on P28. **C)** Quantification of skin pigmentation relative to control. **D)** Representative Fontana & Masson staining of dorsal skin. **E)** Quantification of melanin relative to control. Pigmentation index is calculated as pigmentation area normalised by length of skin. Graphs show mean ± S.E.M. One way ANOVA. ****$p<0.0001$, ns $p>0.05$.

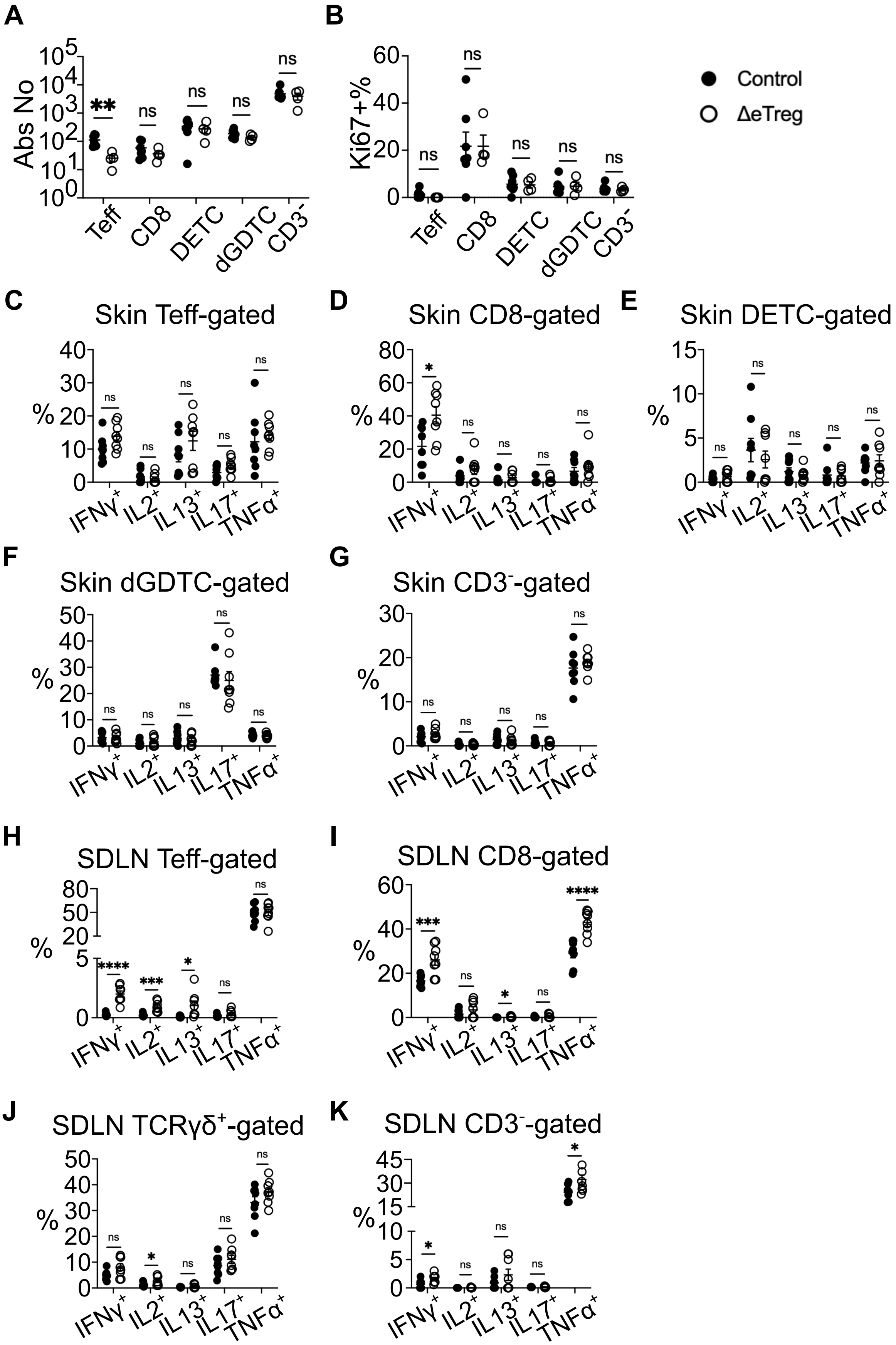

Supplementary figure 6. Early Treg depletion causes inflammatory response in the skin and SDLN. A-B) Flow cytometric quantification of **A)** skin T cell numbers and

B) proliferation as measured by percentage Ki-67 expression. **C) C-K)** Percentage expression of cytokines following stimulation with PMA/Ionomycin. **C-G)** Skin. **C)** Teffs. **D)** $CD8^+$ T cells. **E)** DETCs. **F)** dGDTCs. **G)** $CD3^-$ immune cells. **H-K)** SDLN. **H)** Teffs. **I)** $CD8^+$ T cells. **J)** $TCR\gamma\delta^+$ T cells. **K)** $CD3^-$ immune cells. Data are pooled from two independent experiments. Graphs show mean ± S.E.M (n=4 biological replicates). Unpaired t-test. ****p<0.0001, ***p<0.001, **p<.01, *p<0.05, ns p>0.05.

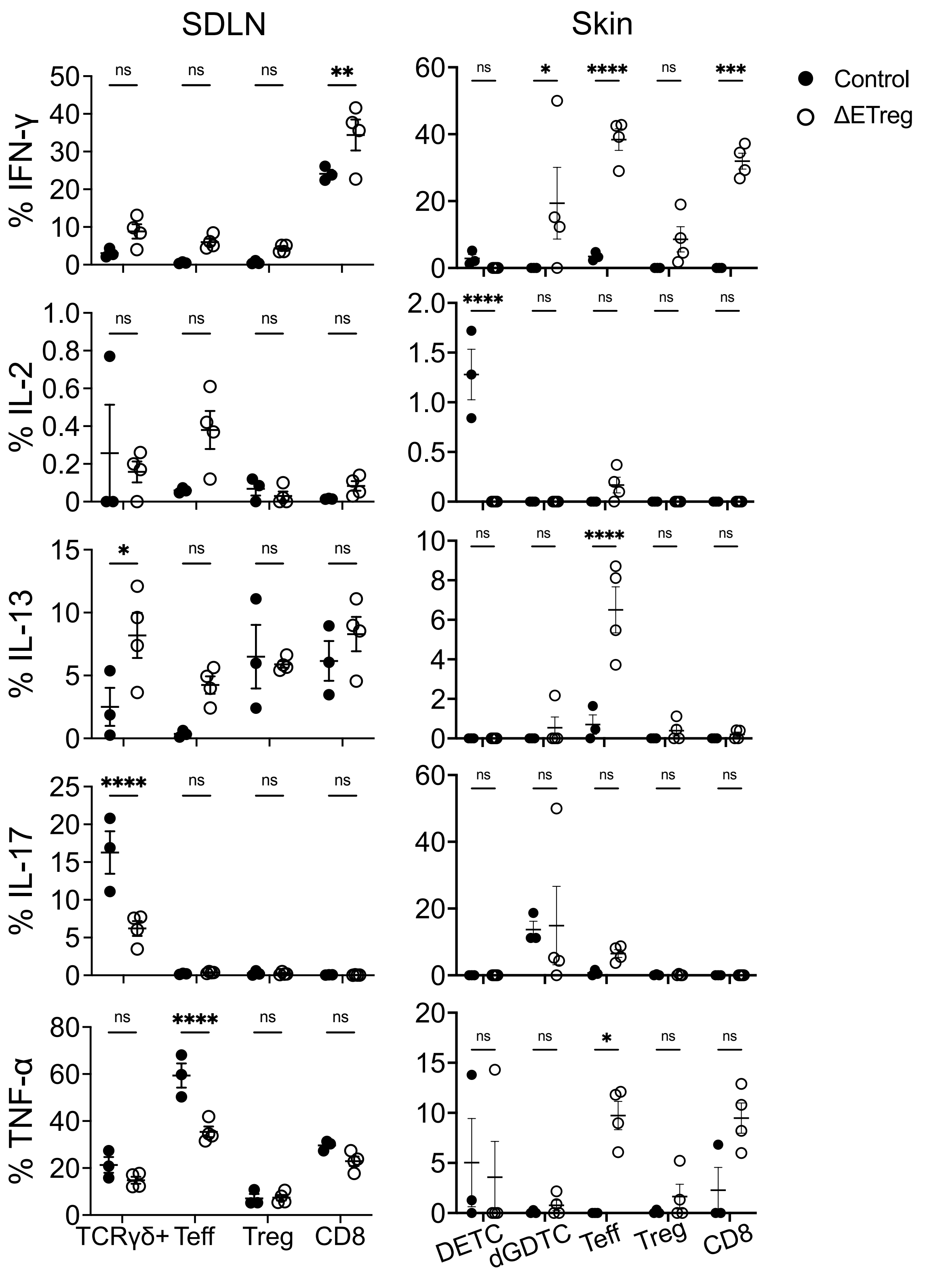

Supplementary figure 7. Increased IFN-γ production on P17 after Treg depletion. Flow cytometric quantification of cytokine production was performed using PMA/Ionomycin-stimulated T cells isolated from skin-draining lymph node (SDLN) or skin. Graphs show mean ± S.E.M (n=3-4 biological replicates). Unpaired t-test. ****p<0.0001, ***p<0.001, **p<.01, *p<0.05, ns p>0.05.

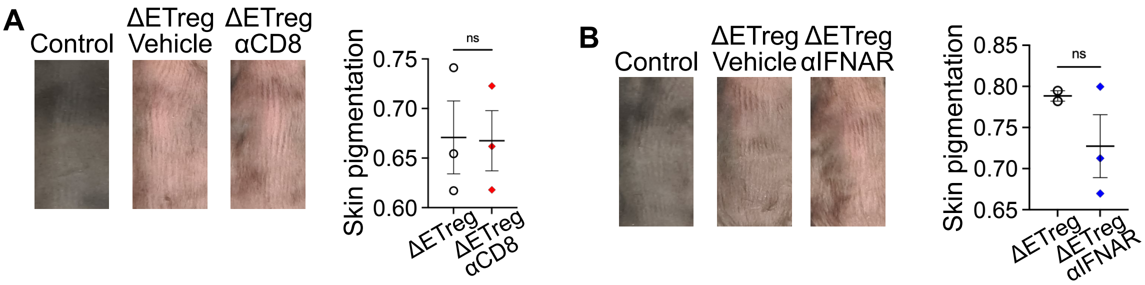

Supplementary figure 8. Modulation of skin pigmentation. A-D) Mice were intraperitoneally injected on P6 and P8. Dorsal skin tissues were harvested and photographed on P28. **A)** *Foxp3-DTR* mice received PBS (control) or DT (ΔEtreg) in addition to PBS (vehicle) or 100 µg of α-CD8 depleting antibody (αCD8) per injection. αCD8 groups received further injections on P15 and P22. **B)** *Foxp3-DTR* mice received PBS (control), or DT (ΔEtreg) in addition to PBS (vehicle) or 100 µg of α-IFNAR blocking antibody (αIFNAR) per injection. Graphs show mean ± S.E.M. Data shows biological replicates (n=2-3). Unpaired t-test. ns $p>0.05$.

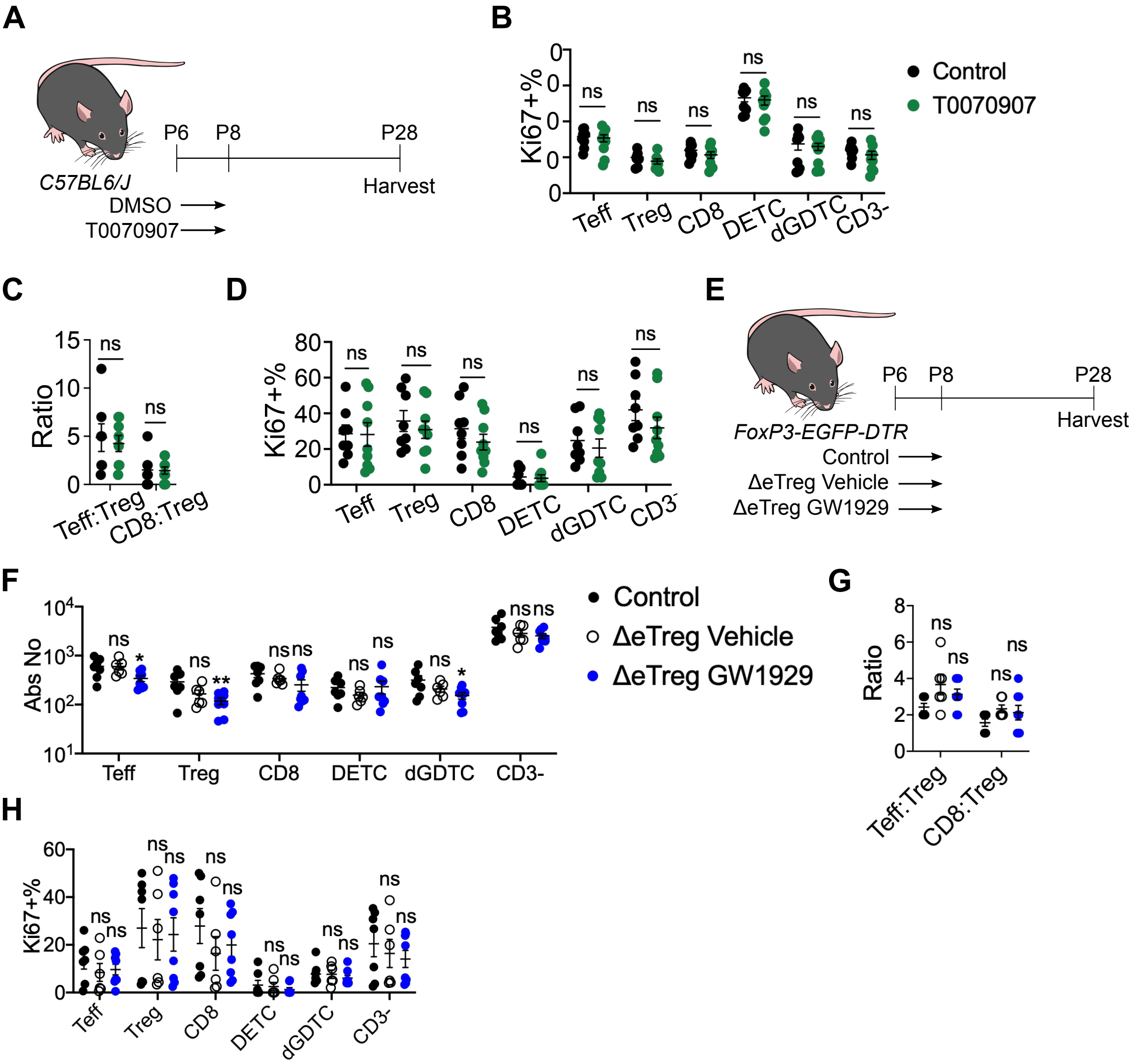

Supplementary figure 9. Modulation of neonatal PPARγ signalling does not affect skin-resident immune cell numbers or proliferation. A) Schematic of experimental timeline. (NIAID Visual & Medical Arts. (10/7/2024). Lab Mouse. NIAID NIH BIOART Source. bioart.niaid.nih.gov/bioart/281). C57BL6/J mice received 2.5 μg/g of T0070907 or DMSO on P6 and P8. Skin tissues were harvested on P28. **B-E)** Flow cytometric quantification of skin-resident T cells and Tregs. **B)** Absolute number. **C)** Teff:Treg and CD8:Treg ratio. **D)** Percentage of proliferating Ki67^{+} cells. **E)** Schematic outline of rescue experiment using PPARγ agonist. (NIAID Visual & Medical Arts. (10/7/2024). Lab Mouse. NIAID NIH BIOART Source. bioart.niaid.nih.gov/bioart/281). Control group received PBS and DMSO on P6 and P8. Treg-depleted ΔEtreg groups received DMSO (vehicle) or GW1929 in addition to DT on P6 and P8. Skin tissues were harvested on P28. **F)** Absolute number. **G)** Teff:Treg and CD8:Treg ratio. **H)** Percentage of proliferating Ki67^{+} cells. Data are pooled from two independent experiments. **B-D, F-H)** Graphs show mean ± S.E.M. (n=4-5 biological replicates). **B-D)** Unpaired t-test. **F-H**) One way ANOVA against control. ns p>0.05.

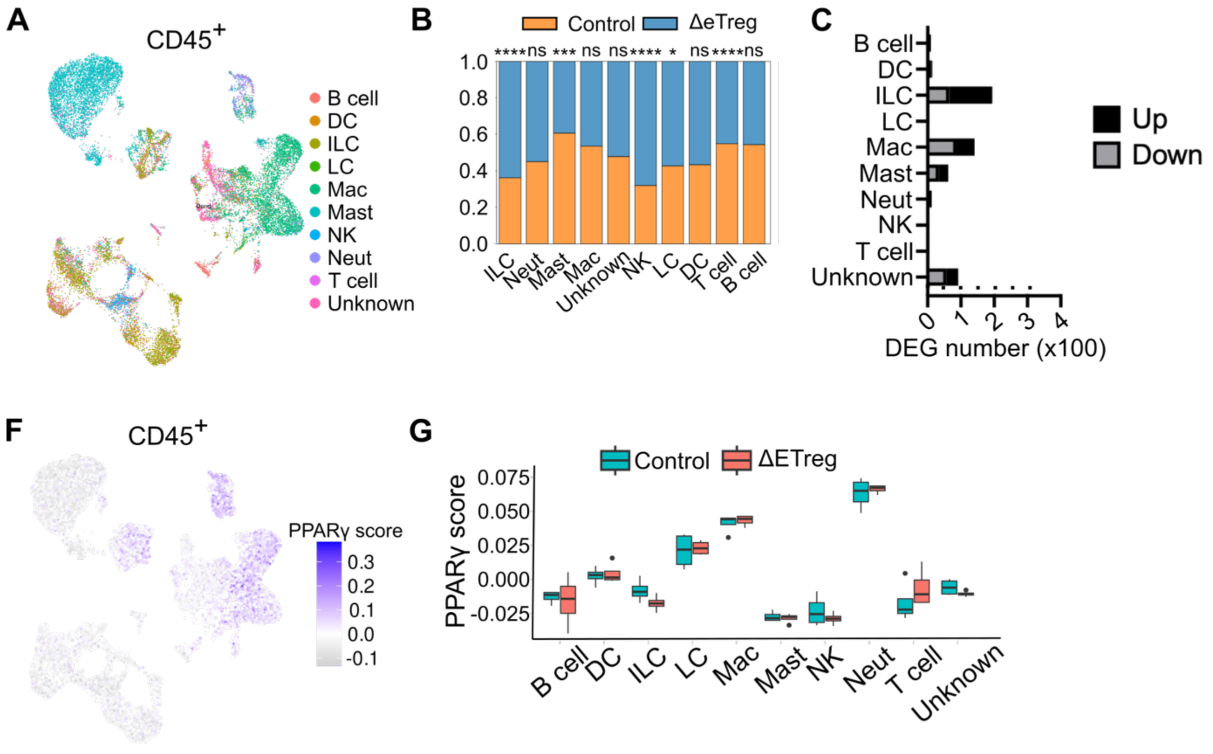

Supplementary figure 10. Immune cells are minimally impacted by early neonatal Treg depletion. A) UMAP of CD45⁺ immune cells. B cell. DC, dendritic cell. ILC, innate lymphoid cell. LC, Langerhans cell. Mac, macrophage. Mast, mast cell. NK, natural killer cell. Neut, neutrophil. T cell. Unknown. **B)** Ratio of immune cells in control and ΔETreg skin. **C)** Number of differentially expressed genes (DEG) by cell type. **F-G)** PPARγ activity score.
