# Supplementary material for: Early skin seeding regulatory T cells modulate PPARγ-dependent skin pigmentation
Source: Nat Commun. 2025 Dec 9;16:11411. doi: 10.1038/s41467-025-66229-2 (PMC12738793; doi:10.1038/s41467-025-66229-2)
Supplement: Supplementary file 2 — Description of Additional Supplementary Files [file 41467_2025_66229_MOESM2_ESM.pdf]

Description of Additional Supplementary Files

File name: Supplementary Data 1

Description: Whole skin bulk RNAseq DEG table.

File name: Supplementary Data 2

Description: $CD45^-$ single cell RNAseq DEG table.

File name: Supplementary Data 3

Description: $CD45^+$ single cell RNAseq DEG table.

File name: Supplementary Data 4

Description: Sorted skin and lymph node Treg DEG table.

File name: Supplementary Data 5

Description: Marker for developing melanocytes.

File name: Supplementary Data 6

Description: List of antibodies used for flow cytometry.
