# Supplementary material for: Early skin seeding regulatory T cells modulate PPARγ-dependent skin pigmentation
Source: Nat Commun. 2025 Dec 9;16:11411. doi: 10.1038/s41467-025-66229-2 (PMC12738793; doi:10.1038/s41467-025-66229-2)
Supplement: Supplementary file 9 — Reporting Summary [file 41467_2025_66229_MOESM9_ESM.pdf]

nature portfolio

Corresponding author(s): Niwa Ali

Last updated by author(s): 11 Sept 2025

Reporting Summary

Nature Portfolio wishes to improve the reproducibility of the work that we publish. This form provides structure for consistency and transparency in reporting. For further information on Nature Portfolio policies, see our Editorial Policies and the Editorial Policy Checklist.

Statistics

For all statistical analyses, confirm that the following items are present in the figure legend, table legend, main text, or Methods section.

n/a	Confirmed	
☐	☒	The exact sample size (*n*) for each experimental group/condition, given as a discrete number and unit of measurement
☐	☒	A statement on whether measurements were taken from distinct samples or whether the same sample was measured repeatedly
☐	☒	The statistical test(s) used AND whether they are one- or two-sided<br>*Only common tests should be described solely by name; describe more complex techniques in the Methods section.*
☐	☒	A description of all covariates tested
☐	☒	A description of any assumptions or corrections, such as tests of normality and adjustment for multiple comparisons
☐	☒	A full description of the statistical parameters including central tendency (e.g. means) or other basic estimates (e.g. regression coefficient) AND variation (e.g. standard deviation) or associated estimates of uncertainty (e.g. confidence intervals)
☐	☒	For null hypothesis testing, the test statistic (e.g. *F*, *t*, *r*) with confidence intervals, effect sizes, degrees of freedom and *P* value noted<br>*Give P values as exact values whenever suitable.*
☒	☐	For Bayesian analysis, information on the choice of priors and Markov chain Monte Carlo settings
☒	☐	For hierarchical and complex designs, identification of the appropriate level for tests and full reporting of outcomes
☒	☐	Estimates of effect sizes (e.g. Cohen's *d*, Pearson's *r*), indicating how they were calculated

Our web collection on statistics for biologists contains articles on many of the points above.

Software and code

Policy information about availability of computer code

Data collection	No code was used to collect the data required for this study
Data analysis	GraphPad Prism 9 and 10 (GraphPad Prism, RRID:SCR_002798) was utilized for statistical and visual output. Single cell RNA-seq analyses were performed using R (vs. 4.2.1) on RStudio (vs. 2022.070.01). No custom code was required and all relevant citations to previously produced code is found in the manuscript.

For manuscripts utilizing custom algorithms or software that are central to the research but not yet described in published literature, software must be made available to editors and reviewers. We strongly encourage code deposition in a community repository (e.g. GitHub). See the Nature Portfolio guidelines for submitting code & software for further information.

Data

Policy information about availability of data

All manuscripts must include a data availability statement. This statement should provide the following information, where applicable:

- Accession codes, unique identifiers, or web links for publicly available datasets
- A description of any restrictions on data availability
- For clinical datasets or third party data, please ensure that the statement adheres to our policy

The scRNA-seq and bulk RNA-seq data described in this manuscript will be deposited at Gene Expression Omnibus and made publicly available upon acceptance of this manuscript.

Research involving human participants, their data, or biological material

Policy information about studies with human participants or human data. See also policy information about sex, gender (identity/presentation), and sexual orientation and race, ethnicity and racism.

Reporting on sex and gender	No human participants were involved in this project
Reporting on race, ethnicity, or other socially relevant groupings	No human participants were involved in this project
Population characteristics	No human participants were involved in this project
Recruitment	No human participants were involved in this project
Ethics oversight	No human participants were involved in this project

Note that full information on the approval of the study protocol must also be provided in the manuscript.

Field-specific reporting

Please select the one below that is the best fit for your research. If you are not sure, read the appropriate sections before making your selection.

[x] Life sciences [ ] Behavioural & social sciences [ ] Ecological, evolutionary & environmental sciences

For a reference copy of the document with all sections, see nature.com/documents/nr-reporting-summary-flat.pdf

Life sciences study design

All studies must disclose on these points even when the disclosure is negative.

Sample size	Sample sizes can all be found in figure legends. Sample sizes for littermate-controlled animal studies were guided by previous studies and were contingent on size of litters. Previous studies that informed sample sizes are as follows:<br>Ali et al. (2017) Cell. doi.org/10.1016/j.cell.2017.05.002<br>Scharschmidt et al. (2015) Immunity. doi.org/10.1016/j.immuni.2015.10.016<br>Boothby et al. (2021) Nature. doi.org/10.1038/s41586-021-04044-7
Data exclusions	No data was excluded from the study
Replication	All experiments were replicated at least three times.
Randomization	Mice were randomly allocated to cages and groups.
Blinding	Researchers were blind to the genotype of the animals (e.g. whether WT control or DTR-positive).

Reporting for specific materials, systems and methods

We require information from authors about some types of materials, experimental systems and methods used in many studies. Here, indicate whether each material, system or method listed is relevant to your study. If you are not sure if a list item applies to your research, read the appropriate section before selecting a response.

Materials & experimental systems

n/a	Involved in the study	
[ ]	[x]	Antibodies
[ ]	[x]	Eukaryotic cell lines
[x]	[ ]	Palaeontology and archaeology
[ ]	[x]	Animals and other organisms
[x]	[ ]	Clinical data
[x]	[ ]	Dual use research of concern
[x]	[ ]	Plants

Methods

n/a	Involved in the study	
[x]	[ ]	ChIP-seq
[ ]	[x]	Flow cytometry
[x]	[ ]	MRI-based neuroimaging

Antibodies

Antibodies used	The following antibodies were used for flow cytometry: anti-CD45-AF700 (Invitrogen, 1/200, cat#56-0451-82, clone 30-F11), anti-CD3-PerCP-Vio700 (Miltenyi, 1/200, cat#130-120-826, clone REA641), anti-TCRγδ-PE-Vio770 (Miltenyi, 1/100, cat#130-123-290,

clone 11F2), anti-CD4-BV650 (Biolegend, 1/200, cat#100545, clone RM4-5), anti-CD8-APC-Vio770 (Miltenyi, 1/50, cat#130-120-0806, clone 53-6.7), anti-CD25-PE-Vio615 (Miltenyi, 1/50, cat#130-123-028, clone 7D4), anti-CD27-BV605 (1/100, BD Biosciences, cat#563365, clone LG.3A10), anti-ICOS-BV711 (Biolegend, 1/100, cat#313547, clone 7E.17G9), anti-Ki67-BV786 (BD Biosciences, 1/100, cat#563756, clone B56), anti-Foxp3-Vio667 (Miltenyi, 1/100, cat#130-111-604, clone FJK-16s), anti-CTLA4-BV421 (Biolegend, 1/100, cat#106311, clone UC10-4B9), anti-CD45-Viogreen (Miltenyi, 1/100, cat#130-110-803, clone 30-F11), CD10a-BV711 (BD Biosciences, 1/200, cat#740740, clone HI10a), anti-Sca1-PE-Vio770 (Miltenyi, 1/200, cat#130-106-220, clone D7), anti-CD34-AF647 (BD Biosciences, 1/100, cat#560230, clone RAM34), anti-CD49f (BD Biosciences, 1/200, cat#563707, clone GoH3), anti-CD117-BV605 (Biolegend, 1/400, cat#135122, clone 2B8).

Validation

Each antibody was titrated and has been widely used in the field. History of citation and validation is available at citeab.com

Eukaryotic cell lines

Policy information about cell lines and Sex and Gender in Research

Cell line source(s)

Melan A mouse melanocyte cell line was a gift from Dr Julian Downward.

Authentication

Cell line was authenticated by their propensity to produce melanin when cultured in vitro with PMA.

Mycoplasma contamination

lines tested negative for mycoplasma

Commonly misidentified lines
(See ICLAC register)

Name any commonly misidentified cell lines used in the study and provide a rationale for their use.

Animals and other research organisms

Policy information about studies involving animals; ARRIVE guidelines recommended for reporting animal research, and Sex and Gender in Research

Laboratory animals

Mus musculus. All mice were maintained on C57BL/6J background

Wild animals

No wild animals were used

Reporting on sex

Experiments were performed using both male and female mice. Individual sex was not recorded.

Field-collected samples

N/A

Ethics oversight

All mouse procedures were approved by local ethical approval at King's College London (UK) (PP70/8474, establishment license X24D82DFF), and performed under a UK Government Home Office license (PP6051479). All methods were carried out in accordance with relevant guidelines and regulations under the UK animals (Scientific procedures) Act 1986, and were reported in accordance with ARRIVE guidelines. All possible efforts were made to minimize animal suffering. All experiments were performed on animals with no prior procedures. Animals were sacrificed using cervical dislocation, followed by permanent cessation of circulation as secondary.

Note that full information on the approval of the study protocol must also be provided in the manuscript.

Plants

Seed stocks

N/A

Novel plant genotypes

N/A

Authentication

N/A

Flow Cytometry

Plots

Confirm that:

- [x] The axis labels state the marker and fluorochrome used (e.g. CD4-FITC).
- [x] The axis scales are clearly visible. Include numbers along axes only for bottom left plot of group (a 'group' is an analysis of identical markers).
- [x] All plots are contour plots with outliers or pseudocolor plots.
- [x] A numerical value for number of cells or percentage (with statistics) is provided.

Methodology

Sample preparation	Single-cell suspensions of full-thickness skin for flow cytometry was performed as previously described. Isolation of cells from axillary, brachial and inguinal lymph nodes for flow cytometry was performed by mashing tissue over 70 µm sterile filters. To prepare dorsal skin cell suspension, shaved mouse skin was de-fatted, minced finely with scissors, and re-suspended in a 3 ml of C10 (RPMI-1640 with L-glutamine with 10% heat-inactivated FBS, 1% penicillin-streptomycin, 1 mM Sodium pyruvate, 1% Hepes, 1x Non-essential amino acid and 60 µM β-mercaptoethanol) supplemented with 2mg/ml collagenase XI, 0.5mg/ml hyaluronidase and 0.1mg/ml DNase in a 50 ml conical. The mixture was digested in a shaking incubator at 37°C at 255 rpm for 45 mins. After a vigorous shaking, the digested mixture was passed through a sterile 100 µm filter fitted onto a 50 ml conical. After pelleting, the filtrate was then filtered once more through a 40 µm strainer fitted onto a new 50 ml conical. Finally, the cells were pelleted once more and re-suspended in 1 ml of C10. Epidermal cell suspensions were prepared by scraping the dermis away with forceps and incubating the layer of epidermis on 3 mL of 0.5% Trypsin-EDTA (ThermoFisher) at 37˚C for 1 hour. Epidermal cells were isolated by scraping the trypsinised epidermis on a petri dish containing 4 ml of C10 media. The mixture of cell suspension and scraped skin was filtered through a 70 µm strainer fitted onto a 50 ml conical. Finally, the cells were pelleted and re-suspended in C10. After cell count using nucleocounter (Chememoetec), cells were plated on nunc round bottom 96-well plates (Thermofisher) for staining.
Instrument	All samples were run on Fortessa LSRII. BD Biosciences.
Software	FlowJo
Cell population abundance	Purity of the sorted Tregs were determined by by analysing the post-sorted cell's percentage expression of EGFP (under Foxp3 locus).
Gating strategy	Gating strategy is available as supplementary figure 1

☒ Tick this box to confirm that a figure exemplifying the gating strategy is provided in the Supplementary Information.
